# Supplementary material for: Novel Rigidochromic and Anti-Kasha Dual Emission Fluorophores Based on D-π-A Dyads as the Promising Materials for Potential Applications Ranging from Optoelectronics and Optical Sensing to Biophotonics and Medicine
Source: Int J Mol Sci. 2023 Mar 18;24(6):5818. doi: 10.3390/ijms24065818 (PMC10057995; doi:10.3390/ijms24065818)
Supplement: Supplementary file 1 [file ijms-24-05818-s001.zip › ijms-2090890-supplementary.pdf]

Supporting Information

Article

Novel rigidochromic and anti-Kasha (DE) fluorophores based on D-π-A diads as the promising materials for potential applications ranging from optoelectronics and optical sensing to biophotonics and medicine

Svetlana A. Lermontova[1], Maxim V. Arsenyev[1], Anton V. Cherkasov[1], Georgy K. Fukin[1], Andrey V. Afanasyev[2], Andrey V. Yudintsev[3], Ilya S. Grigoryev[1], Elena Yu. Ladilina[1], Tatyana S. Lyubova[1], Natalia Yu. Shilyagina[3], Irina V. Balalaeva[3], Larisa G. Klapshina[1] and Alexandr V. Piskunov[1,*]

* Correspondence: pial@iomc.ras.ru; Tel.: +7 920 045 18 04

Table S1.

Crystal data and structure refinement details for **AntTCNE** and **PyrTCNE**.

	**AntTCNE**	**PyrTCNE**
T	298 K	100 K
Empirical formula	$C_{19}H_9N_3$	$C_{21}H_9N_3$
M	279.29	303.31
Crystal system	Monoclinic	Monoclinic
Space group	$P2_1/c$	$P2_1/n$
Unit cell dimensions	a = 7.0957(4) Å	a = 7.8371(8) Å
	b = 10.5265(6) Å	b = 7.4670(9) Å
	c = 20.0061(12) Å	c = 24.535(3) Å
	α = 90°	α = 90°
	β = 90.020(2)°	β = 92.972(4)°
	γ = 90°	γ = 90°
V, Å^3	1494.31(15)	1433.9(3)
Z	4	4
d_{calc}, g/cm^3	1.241	1.405
μ, mm^{-1}	0.076	0.085
F_{000}	576	624
Crystal dimensions, mm	0.50 × 0.23 × 0.08	0.77 × 0.25 × 0.24
θ range for data collection, °	2.04-25.09	2.49-27.16
HKL indicies	$-8 \le h \le 8$	$-10 \le h \le 10$
	$-12 \le k \le 12$	$-9 \le k \le 9$
	$-23 \le l \le 23$	$-31 \le l \le 31$
Reflns collected	18766	15074
Independent reflns with $I>2\sigma(I)$ [R_{int}]	1582 [0.0550]	2400 (0.1478)

Completeness to θ, %	97.6	99.5
Data / restraints / parameters	2600 / 299 / 277	3208 / 0 / 219
$S(F^2)$	1.053	1.038
Final R indices ($F^2>2\sigma(F^2)$)	R_1 = 0.0663	R_1 = 0.0804
	wR_2 = 0.1163	wR_2 = 0.2013
R indices (all data)	R_1 = 0.1180	R_1 = 0.1088
	wR_2 = 0.1347	wR_2 = 0.2179
Largest diff peak and hole, e/Å^3	0.11 / -0.12	0.36 / -0.43

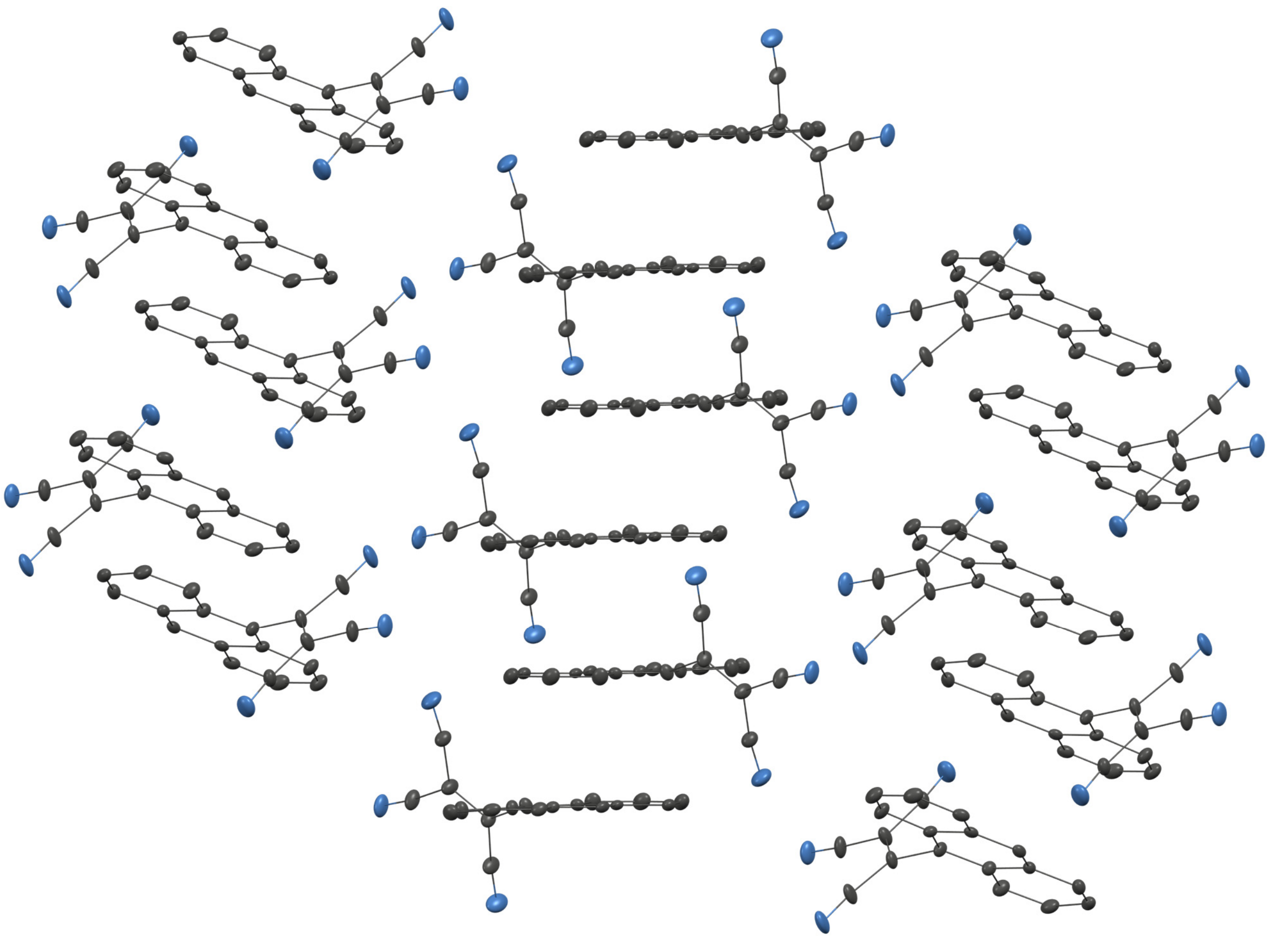

Figure S1. The fragment of the crystal packing of **AntTCNE** along the *(-1 4 -2)* vector. Thermal ellipsoids are drawn at 10% probability level. Hydrogen atoms are omitted for clarity. Color code: carbon, black; nitrogen, blue.

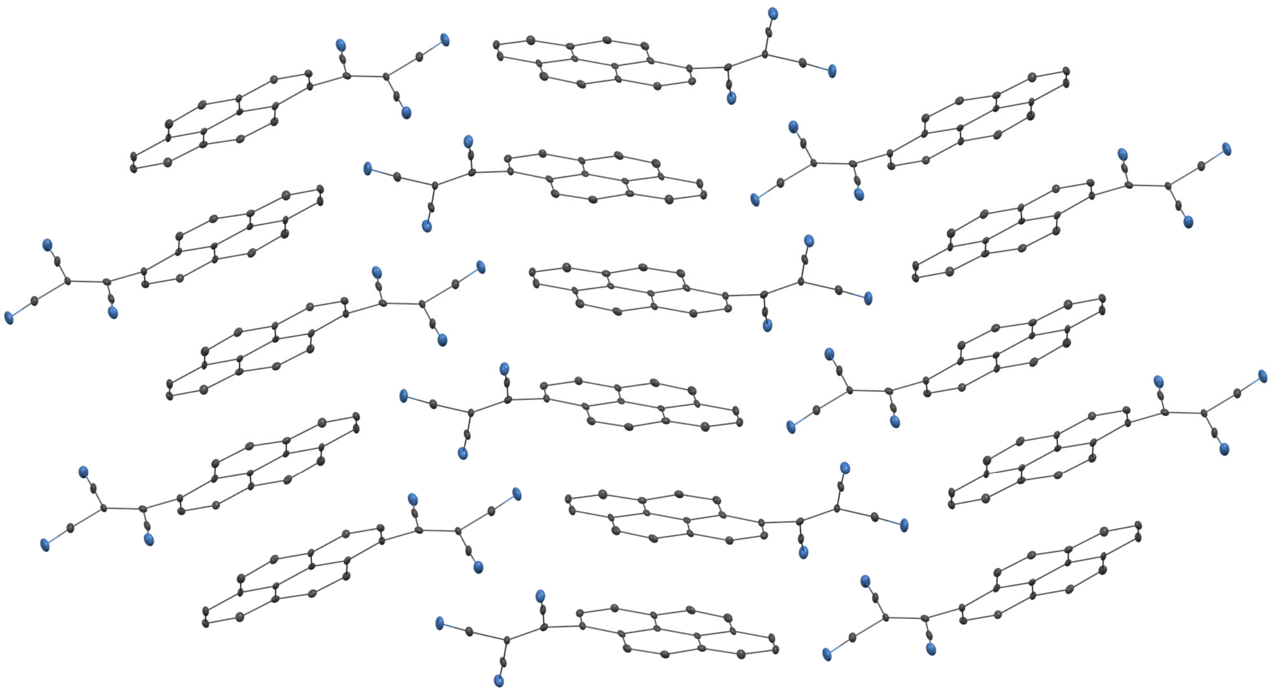

Figure S2. The fragment of the crystal packing of **PyrTCNE** along the *(3 0 0)* vector. Thermal ellipsoids are drawn at 10% probability level. Hydrogen atoms are omitted for clarity. Color code: carbon, black; nitrogen, blue.

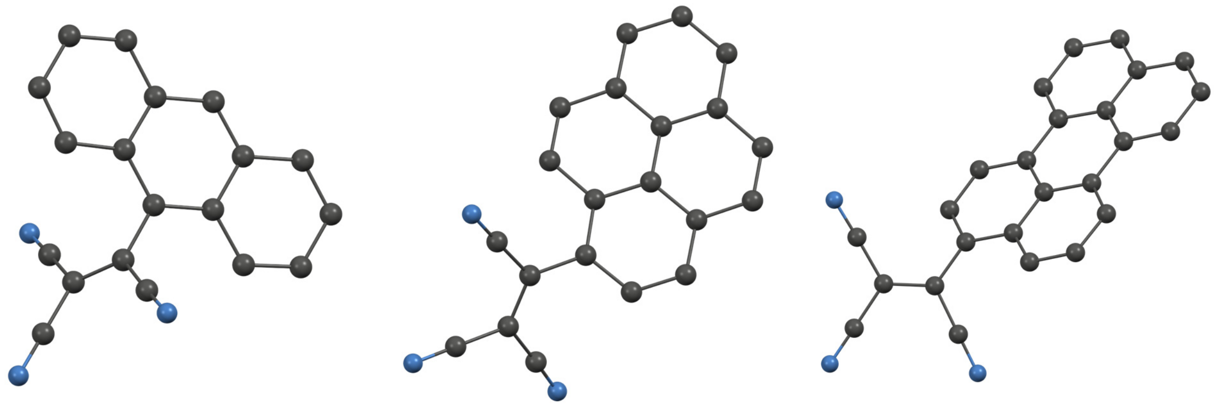

Figure S3. Theoretical molecular structures of **AntTCNE** (left), **PyrTCNE** (center) and **PerTCNE** (right). Hydrogen atoms are omitted for clarity. Color code: carbon, black; nitrogen, blue.

Table S2.

Selected bond lengths in **AntTCNE**, **PyrTCNE** and **PerTCNE**.

	**AntTCNE**	**PyrTCNE**	**PerTCNE**
N(1)-C(1)	1.116(5)<br>[1.161]	1.144(5)<br>[1.161]	-<br>[1.162]
N(2)-C(3)	1.118(5)<br>[1.161]	1.141(4)<br>[1.161]	-<br>[1.162]
N(3)-C(5)	1.117(5)<br>[1.162]	1.142(4)<br>[1.161]	-<br>[1.161]
C(2)-C(1)	1.451(6)<br>[1.431]	1.432(5)<br>[1.430]	-<br>[1.430]
C(2)-C(3)	1.450(6)<br>[1.431]	1.439(4)<br>[1.431]	-<br>[1.430]
C(4)-C(5)	1.452(6)<br>[1.431]	1.449(5)<br>[1.434]	-<br>[1.434]
C(2)-C(4)	1.335(5)<br>[1.368]	1.364(4)<br>[1.375]	-<br>[1.377]
C(4)-C(6)	1.520(5)<br>[1.481]	1.474(4)<br>[1.470]	-<br>[1.457]

The theoretical data are given in square brackets. For **AntTCNE**, the bond lengths of only one of the TCNE sites are given.

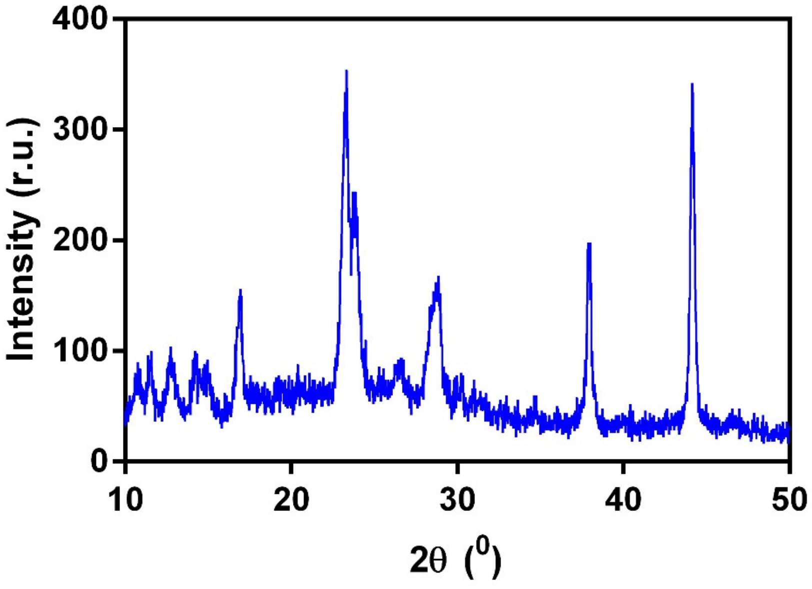

Figure S4. X-ray diffraction pattern of **PerTCNE** powder.

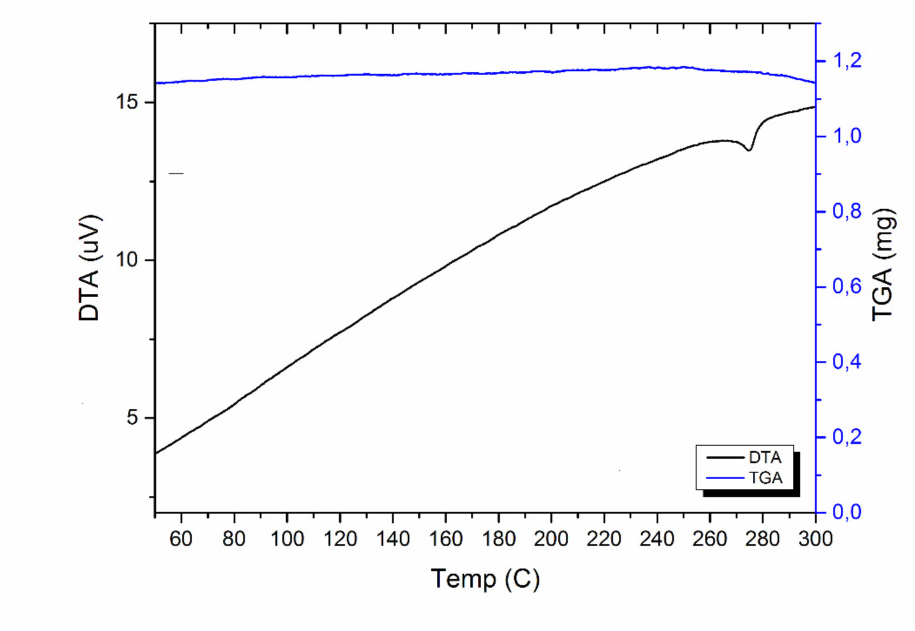

Figure S5. Thermogravimetric and differential thermal analysis curves of **PerTCNE**.

Table S3. Molar extinction coefficients for AntTCNE, PyrTCNE and PerTCNE in CH_3CN

Compound	Wavelength	lg ε
AntTCNE	369	3,75
	523	3,28
PyrTCNE	326	4,07
	500	3,77
PerTCNE	416	3,84
	570	3,79

The calculation of optical density

Optical density D was calculated using decimal logarithm of cuvette transmittance T. D=2-log(T,%). D additional corresponds to difference between D and final experimental D at the largest time at the last point (approx. 215 hours).
D is proportional to concentration of the molecules so time dependence demonstrates concentration decay as well.

Table S4. Decay time τ:

Irradiation fluence, J/cm^2	Decay time, h
30	102
120	75

Decay time τ was calculated assuming $D=D_0*\exp(-t/\tau)$ dependence as the best suitable.
For all graphs the correlation coefficient is not lower than 0.96.

Fluorescence quantum yield measurements [1]
Fluorescence quantum yields (Φ_s) are reported relative to cresyl violet perchlorate in MeOH ($\Phi_r = 0.54$)[1]. The experiments were performed using optically matched solutions and the quantum yields were calculated using Equation S1,

$$\Phi_s = \Phi_r \cdot \frac{A_r F_s}{A_s F_r} \cdot \frac{n_s^2}{n_r^2} \text{ (Equation S1)}$$

where, A_s and A_r are the absorbance of the sample and reference solutions, respectively, at the same excitation wavelength; F_s and F_r are the corresponding relative integrated fluorescence intensities of the sample and the reference; and n_s and n_r is the refractive index of the solvent used to measure the sample and the reference.

[1] .J.R. Lakowicz, Principiles of Fluorescence Spectroscopy, 1999.

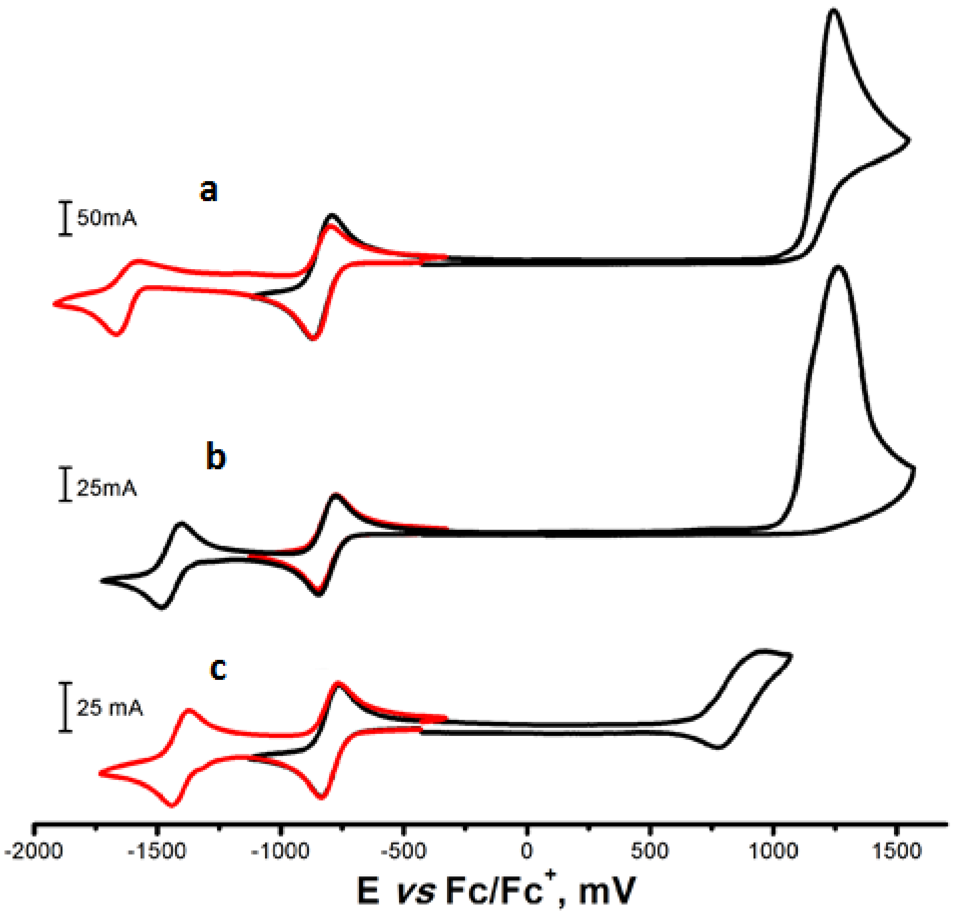

Figure S6. Cyclic voltammograms of **AntTCNE** (**a**), **PyrTCNE** (**b**) and **PerTCNE** (**c**)

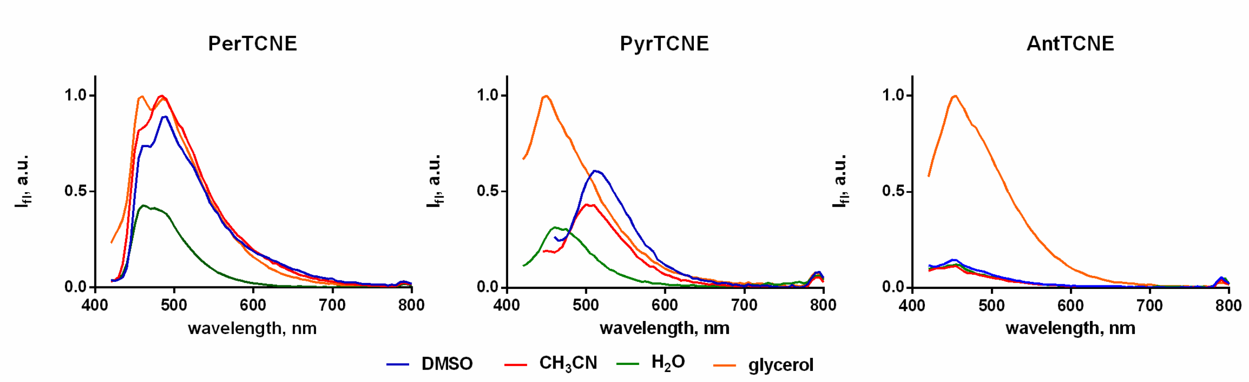

Figure S7. Emission spectra of **AntTCNE** (**a**), **PyrTCNE** (**b**) and **PerTCNE** (**c**) in the different solvent (10^{-6} mol/L), λ_{ex} = 400 nm.

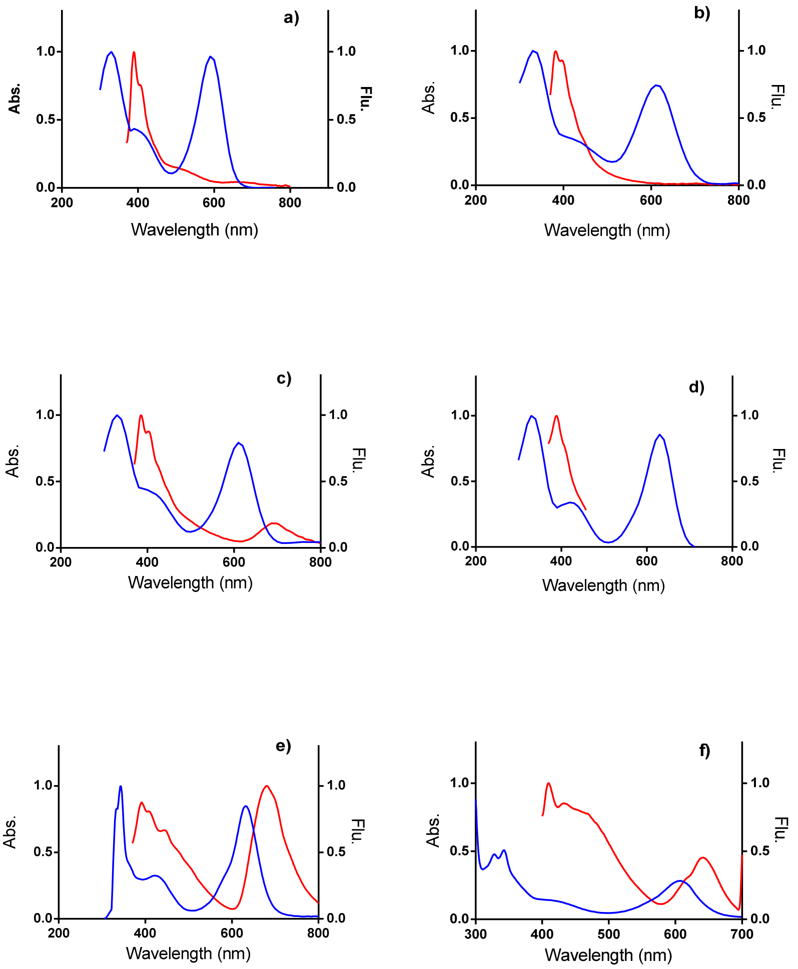

Figure S8 Normalized spectra of absorption and fluorescence of of **Pyr4CN4Pz** (λ_{exit} = 350 nm, 10^{-6} mol/L), according to Levshin's law [2:]; **(a)** – CH_3CN**, (b)** – H_2O **(c)** – $PhCH_3$, **(d)** – THF, **(e)** – castor oil, **(f)** – polymer film

[2] Andrii V. Kulinich, Alexander A. IshchenkoStanislav L. BondarevValery N. Knyukshto Effect of donor and acceptor end-groups on electronic structure and spectral-fluorescent properties of merocyanines in frozen ethanol. Journal of Photochemistry & Photobiology, A: Chemistry 405 (2021) 112932 https://doi.org/10.1016/j.jphotochem.2020.112932

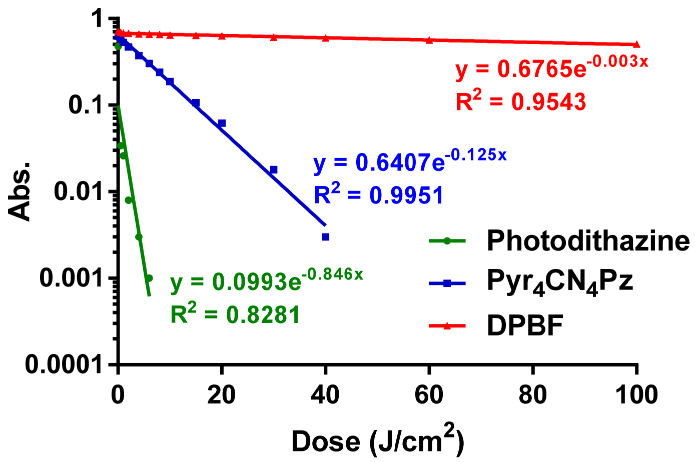

Figure S9. DPBF photobleaching sensitized by **Pyr_4CN_4Pz** and Photoditazine in DMSO under light irradiation (615–635 nm, 20 mW/cm^2) and measured by absorption at 420 nm.

The quantum yield of singlet oxygen generation was calculated relative to the quantum yield of the Photodithazine (0.56 [1]) using the Equation:

$$\varphi_{\Delta 1} = \frac{\varphi_{\Delta 0} \times k_1 \times D_0}{k_0 \times D_1},$$

where $\varphi_{\Delta 0}$ is the quantum yield of singlet oxygen generation of Photodithazine; $\varphi_{\Delta 1}$ is the quantum yield of singlet oxygen generation of tested compound; k_1 and k_0 are rate constants of DPBP trap photobleaching sensitized by tested compound and Photodithazine, respectively; D_1 and D_0 are optical density of the compound and Photodithazine (without adding a trap DPBF).

1. Bagrov, I.V., Dadeko, A., Kiselev, V.M., Muravieva, T.D., Starodubtsev, A.M., 2019. Comparative studies of the photophysical properties of dimegin, photoditazine and radachlorin. Journal of Technical Physics 126, 162.

.

Table S5. Optical density values at 540 nm for DMSO and cells treated with Pyr_4CN_4Pz without the addition of MTT reagent

	OD_{540}*
DMSO	0.039 ± 0.003
Cells with 5 uM Pyr_4CN_4Pz	0.041 ± 0.003
Cells with 10 uM Pyr_4CN_4Pz	0.042 ± 0.003
* mean OD values and SD are indicated	

In order to evaluate the possible effect of Pyr_4CN_4Pz absorption at 540 nm on the results of the MTT assay, we checked the applicability of the protocol prior to the start of the experiment. To do this we performed the whole procedure on the cell culture with exception of the MTT reagent addition: incubation with the photosensitizer at the highest concentrations, changing the medium, light irradiation, incubation for a day, changing the medium again, incubation for four hours, discarding the medium, and dissolution in DMSO. As a result, the optical density in the plate wells did not differ from the blank (DMSO only). For comparison, when conducting the MTT assay, the optical density values reached about 0.5 in wells with control cells.
